# The effect of air pollution quality on lung cancer rates in middle-income and high-income countries: a panel data analysis approach

**DOI:** 10.3389/fpubh.2024.1372320

**Published:** 2024-08-21

**Authors:** Mehmet Gozlu, Osman Senol, Umit Cirakli, Huseyin Aslan, Fevzi Akbulut, Durmus Gokkaya

**Affiliations:** ^1^Institute of Health Sciences, Gaziantep University, Gaziantep, Türkiye; ^2^Department of Health Management, Faculty of Health Sciences, Karadeniz Technical University, Trabzon, Türkiye; ^3^Department of Health Management, Faculty of Health Sciences, Izmir Bakircay University, Izmir, Türkiye; ^4^Health Institutions Management, İzmir Kavram Vocational School, İzmir, Türkiye; ^5^Department of Health Management, Faculty of Health Sciences, Bingol University, Bingöl, Türkiye; ^6^Department of Health Management, Faculty of Economics and Administrative Sciences, Yozgat Bozok University, Yozgat, Türkiye

**Keywords:** lung cancer, air pollution, public health, Driscoll-Kraay standard error approach, panel data analysis

## Abstract

**Background:**

Air pollution is one of the biggest problems in societies today. The intensity of indoor and outdoor air pollutants and the urbanization rate can cause or trigger many different diseases, especially lung cancer. In this context, this study's aim is to reveal the effects of the indoor and outdoor air pollutants, and urbanization rate on the lung cancer cases.

**Methods:**

Panel data analysis method is applied in this study. The research includes the period between 1990 and 2019 as a time series and the data type of the variables is annual. The dependent variable in the research model is lung cancer cases per 100,000 people. The independent variables are the level of outdoor air pollution, air pollution level indoor environment and urbanization rate of countries.

**Results:**

In the modeling developed for the developed country group, it is seen that the variable with the highest level of effect on lung cancer is the outdoor air pollution level.

**Conclusions:**

In parallel with the development of countries, it has been determined that the increase in industrial production wastes, in other words, worsening the air quality, may potentially cause an increase in lung cancer cases. Indoor air quality is also essential for human health; negative changes in this variable may negatively impact individuals' health, especially lung cancer.

## 1 Introduction

Environmental Protection Agency (EPA) defines the air pollution as “the presence of pollutants in the air in such a way as to adversely affect human health or cause other harmful environmental effects” (1). Air pollution is one of the world's leading health risks. Air pollution ranks fourth in terms of fatal health risks, after metabolic risks, nutritional risks, and smoking (2). The first air pollution records were found in Egypt in the twelfth century. With industrialization in the eighteenth and nineteenth centuries, air pollution entered the global agenda, and in the twentieth century, the factors that caused air pollution were identified (3). Population growth, urban expansion, traffic intensification, and industrial development all contribute to an increase in air pollution, and it is believed that the effects of the content of inhaled air are increasing. In addition, traffic, transport, industry, and heating pollutants are also significant contributors to air pollution (4). For example, in 1931, it is known that 9 days of fog in the Manchester and Salford regions of England, where industry is dense, caused 592 deaths (5). Air pollution, one of the major problems of the last century, causes many health problems. Meeting the growing demand for energy from fossil fuels such as oil and coal due to industrialization and motor vehicles are just some of the leading causes of increasing pollution (6, 7).

According to the World Health Organization's 2019 data, cardiovascular diseases, cancer, and respiratory diseases are the leading causes of death worldwide (8). It is known that the development of lung cancer increases with both the direct effect and the chronic inflammatory effect of carcinogens contained in air pollution (6). For example, a study of 500,000 adults in the USA found that an increase of 10 μm/m^3^ in PM-2.5 concentration increased the incidence of lung cancer by 14% (9). Air pollution is also known to increase the risk of developing lung cancer by 20–30%, according to the results of many cohort and case-control studies (10, 11). Air pollution (indoor and outdoor air pollution) is among the leading risks of premature mortality (8, 12). 7.8% of deaths are attributed to outdoor air pollution (13) and 4.1% to indoor air pollution (14). Indoor air pollution is caused by households burning solid fuel sources such as firewood, crop waste, and fertilizers for cooking and heating. Outdoor air pollution consists of a mixture of pollutants from diverse sources such as transportation, wind-blown dust, burning of biomass, industrial sources, and coal for household energy, and it is generally expressed with the term of “particulate matter”. The World Health Organization states indoor air pollution is “the world's greatest environmental health risk” (15, 16). On the other hand, outdoor air pollution is one of the world's most significant health risk factors caused by air pollutants from industrial waste.

Cancer is the second most important cause of death. Lung cancer is one of the most common types of cancer (15, 16). There are many studies on the relationship between air pollution and lung cancer (17, 18). Information shows that air pollution increases lung cancer in Europe (19). In addition to studies suggesting that a large proportion of lung cancer is tobacco-related, the causes of lung cancer cannot be evaluated only in a narrow framework, such as tobacco use or air pollution (19–21). Field and Withers discussed the causes of lung cancer as radiation, chemicals and mixtures, occupations, metals, dust and fibers, personal habits, and other exposures. Tobacco use is one of the factors within the scope of personal habits and other exposures. Many factors, such as air polluted by coal and domestic fuel, exhaust gas, soot in the air, sulfur mustard, radiation, carcinogenic metals to which the respiratory system is exposed, occupations that require exposure to carcinogenic substances, all forms of asbestos can be counted among the causes of lung cancer (22–24). When we look at the causes of lung cancer mentioned above, it is seen that these factors are environmental factors that indicate that they are present in the air more than the required rates and that air pollution is intense. It is a known fact that these factors are related to countries' development and urbanization rates. The incidence of lung cancer varies between regions (13, 14, 23, 24).

In this context, this study examined the effects of air pollution and urbanization rate on lung cancer. The study's aim was to find out the effects of indoor air pollution, outdoor air pollution, and urbanization rate on the lung cancer cases in middle and high-income countries.

The study is expected to raise awareness of environmental and public health policies, particularly among policymakers in developed and developing countries with high-income levels and high levels of urbanization. When the studies in the literature on the subject are analyzed, it is seen that many of the studies are cohort-type and ecological studies, and these studies were use simple correlation and regression analyzes. But it will be yielding spurious regression to analyze data with time series by simple linear regression. On the other hand, if data have both time and unit dimensions, which means panel data, standard regression analyze will not be enough to get robust evidence about the problem even if the direction of the relationship is correctly revealed. In standard regression and correlation analyses used in most of the studies about the relationship between lung cancer and air pollution, unobserved effects are included in the error term. But data with both time series and cross section should be analyzed with an appropriate analysis that takes into account both the characteristics of the unit and the differences between units. Although the relationship between air pollution and lung cancer is a much-studied subject, it is seen that the number of studies that take these factors into consideration is limited. Therefore, the main motivation behind this study is to provide more robust evidence about the relationship between lung cancer and air pollution. On the other hand, another difference of this research from the others is that it is more comprehensive and deals with the issue at the macro level. Therefore, this study will be able to provide more important evidence for generalization. In addition, since the data set of the research has a time series and cross-sectional dimension, the method used in the research contributes to obtaining more robust evidence. One of the most important outputs of the research is to develop a macro perspective on the relationship between air pollution and lung cancer, which is one of the diseases that directly affect the health level and health expenditure amounts of the society. By determining the effect of air quality on different diseases, especially lung cancer, policies can be developed to limit outdoor and indoor air pollutant particles. Finally, since this research reveals the direct effect of air quality on human health, especially cancer, it contributes to raising awareness as well as providing information to the society.

## 2 Literature review

Cohen and Pope analyzed various studies investigating the relationship between lung cancer and air pollution (25). They focused on case-control and cohort studies, including three prospective cohort studies, which examined the relationship between lung cancer and air pollution in terms of urban and rural populations, reviewed for occupational groups, and read between populations. They also investigated the ambient air pollution-lung cancer effect of the relative risks of various types of exposure to combustion-derived pollutants. They found that combustion-derived air pollution contributes to the occurrence of lung cancer in the general population. They stated that these results are consistent with studies on people exposed to combustion-induced pollution, such as occupational exposure and exposure to environmental tobacco smoke.

Raaschou-Nielsen et al. aimed to evaluate the association between long-term exposure to ambient air pollution and lung cancer incidence in European populations. In this prospective analysis of data from the European Cohort Study on the Effects of Air Pollution, they used 17 cohort studies conducted in nine European countries (16). As a result of this extensive study, they found an association between exposure to particulate matter air pollution and the incidence of lung cancer, especially adenocarcinoma, in Europe.

Tseng et al. investigated the effects of tobacco use and changes in particulate matter levels on lung cancer between Northern Taiwan and Southern Taiwan in Taiwan (19). They analyzed 371,084 patients with lung cancer to assess the prevalence of smoking and correlations between the incidence of adenocarcinoma lung cancer (AdLC) and non-AdLC lung cancer. As a result of the study, they found that more than half of the patients with lung cancer had never smoked and stated that changes in air pollution levels affected the incidence of AdLC and patient survival.

Hu et al. examined the relationship between breast and cervical cancer prevalence in Chinese women and air pollution with panel data analysis (26). They used data from 31 provinces and cities between 2006 and 2015. As a result of the study, they found that the relationship between soot and dust emissions and the prevalence of breast or cervical cancer from 2006 to 2015 was not linear. They also stated that there was an inverted *U*-shaped relationship between the prevalence of breast cancer and cervical cancer and air pollutants between 2006 and 2015.

Pang et al. investigated the spatial, moderating, and equal effects of green areas on lung cancer incidence in air pollution using 3-year lung cancer data from 228 provinces in China (27). Using spatial econometric and threshold models, they found that green spaces reduce the incidence of lung cancer in both local and neighboring provinces. They also stated that when exceeding a certain threshold, there is a reduction in the harmful effect of air pollution on lung cancer incidence in areas with more green areas.

In their experimental toxicology studies, Nesnow and Lewtas documented the mutagenic and carcinogenic properties of combustion-induced air pollution, such as diesel exhaust, which is ubiquitous in urban and motorway environments (28). In addition, Huang et al. found that exposure to green space is protective against lung cancer, and Kayyal-Tarabeia et al. found a beneficial correlation between more green space in dwellings and lower incidence of lung cancer in their study with 144,427 participants (29, 30). Similarly, Zhu et al. examined the relationship between tuberculosis and air pollution, Kasdagli et al. and Crouse et al. examined the relationship between cardiovascular mortality and air pollution (31, 32).

## 3 Materials and methods

### 3.1 Purpose of the study

Air pollution is one of the biggest problems in societies today. The factors causing air pollution are divided into two as internal pollutants caused by domestic wastes and external pollutants caused by different sources like transportation, wind-blown dust, burning of biomass, industrial sources, and coal for household energy. In this study, indoor and outdoor air pollution levels are defined as the annual average concentration of fine suspended particles < 2.5 microns in diameter due to pollutants in the indoor and outdoor environment. On the other hand, the rate of urbanization is an indicator that has a very close relationship with air pollution. The intensity of indoor and outdoor air pollutants and the urbanization rate can cause or trigger many different diseases, especially lung cancer. In this context, this study aims to examine the effect of indoor and outdoor air pollutants and urbanization rates on lung cancer cases.

#### 3.1.1 Universe and sample of the study

While determining the population of the research, the World Bank's classification of countries according to their income status was taken into consideration. The research population consists of high and middle-income group countries. In the middle-income group, data from 25 countries were accessed, and there are 725 observation values in the model produced. In the high-income group, data from 38 countries were obtained, with 1,102 observation values in total. Low-income countries could not be included in the study due to inaccessibility of the data.

### 3.2 Model and data

To be able to generalize the results of the research as much as possible, all countries for which data are available are included in the analysis. A single dependent variable was used in the models developed for both country groups, while three independent variables were used. The lung cancer cases per 100,000 people is included as the dependent variable in the model. The independent variables are the level of outdoor air pollution, the level of indoor air pollution, and the rate of urbanization in the countries. Econometric models will be developed for the effect of the independent variables used within the scope of the research on lung cancer incidence. For both models produced, the period between 1990 and 2019 is taken as the time series dimension of the study, and the data type of variables is annual. Panel data analyses include both cross-sectional and time series in their structure. It is known that some problems may be encountered when different variables with a particular time dimension come together. These problems are multicollinearity, autocorrelation, horizontal cross-section dependence problems, and changing variance problems. After defining the variables and creating the equation, a detailed examination was made to determine whether these problems exist. Data on the variables of the countries selected within the scope of the research were obtained from The Organization for Economic Co-operation and Development (OECD) database. The variables to be used in both models are shown in Table 1.

**Table 1:** Variables and abbreviations.

**Variables**	**Abbreviated symbol**
Lung cancer cases per 100,000 people	Lung cancer
Urbanization rate	Urban
Outdoor air pollution level	Outdoor
Indoor air pollution level	Indoor

As part of the research, an econometric model was developed for each income group of the countries. The models planned to be created within the scope of the research are given below.

Model 1 (Middle-Income Countries):


$$\begin{array}{c} \Delta {LNCancer}_{it}=\;C+\;\overset{pi}{\underset{j=1}{\sum }}\lambda_{ij}\;\Delta LNUrban_{i,t-j}+\\ \overset{qi}{\underset{j=0}{\sum }}\;\delta_{ij}\;\Delta LNOutdoor_{i,t-j}+\;\overset{qi}{\underset{j=0}{\sum }}\varphi_{ij}\;\Delta LNIndoor_{i,t-j}+\;\varepsilon_{it} \end{array}$$

Model 2 (High-Income Countries):


$$\begin{array}{c} \Delta {LNCancer}_{it}=\;C+\;\overset{pi}{\underset{j=1}{\sum }}\lambda_{ij}\;\Delta LNUrban_{i,t-j}+\\ \overset{qi}{\underset{j=0}{\sum }}\;\delta_{ij}\;\Delta LNOutdoor_{i,t-j}+\;\overset{qi}{\underset{j=0}{\sum }}\varphi_{ij}\;\Delta LNIndoor_{i,t-j}+\;\varepsilon_{it} \end{array}$$

Abbreviations included in the equation developed for both country groups: cancer; Lung Cancer Cases per 100,000 people, urban; Urbanization Rate, Outdoor; Outdoor Air Pollution Level, Indoor; Indoor Air Pollution Level definable. The dependent variable is on the left sides of the equations. On the right side of the equations, “c” is included as the constant term, “ε” as the error term, “i” as the unit number in panel, “t” as time, “Δ” as the first differencing operator, and “L” as the natural logarithm of series. When doing panel data modeling, exact estimation of the dependent variable is not possible as there are many factors affecting the dependent variable. However, the effects the other possible variables affecting it is contained within the error term.

## 4 Results

Within the scope of the research, tests regarding the horizontal cross-sectional dependence of the variables or series should be applied before applying the unit root test. Accordingly, Breusch-Pagan CDLM1 and Pesaran CDLM2 and CDLM tests were used to test the variables. In the Table 2, horizontal cross-section dependence tests were applied to the variables. The results of unit root test about whether the series are stationary or not are shown in the Table 3.

**Table 2:** Cross-sectional dependence results of variables.

		**Lung cancer**		**Outdoor**		**Indoor**		**Urban**	
	**Tests**	**Statistics**	**Probability**	**Statistics**	**Probability**	**Statistics**	**Probability**	**Statistics**	**Probability**
Model 1. Middle income group countries	Breusch-Pagan	4,343.3	< 0.001	542.30	< 0.001	662.48	< 0.001	6,779.6	< 0.001
	Pesaran Scaled LM	165.06	< 0.001	9.89	< 0.001	14.79	< 0.001	264.52	< 0.001
	Pesaran CD	34.97	< 0.001	5.01	0.004	5.86	< 0.001	55.04	< 0.001
Model 2. High income group countries	Breusch-Pagan	1,531.4	< 0.001	1,363.3	< 0.001	1,609.74	< 0.001	1,558.60	< 0.001
	Pesaran Scaled LM	341.10	< 0.001	339.82	< 0.001	417.87	< 0.001	392.50	< 0.001
	Pesaran CD	81.70	< 0.001	51.76	< 0.001	122.23	< 0.001	44.09	< 0.001

**Table 3:** CADF panel unit root test.

**Models**	**Variables**	***t*-bar**	**Cv10**	**Cv5**	**Cv1**	***Z*(*t*-bar)**	***P***
***t*****-bar test, N, T** = **(25, 30) Obs** = **650 Augmented by 2 lags (average)**							
Model 1. Middle income group countries	Lung cancer	−2.567	−2.070	−2.150	−2.300	−4.169	< 0.001
	Urban	−2.135	−2.070	−2.150	−2.300	−1.962	0.025
	Outdoor	−2.966	−2.070	−2.150	−2.300	−6.203	< 0.001
	Indoor	−1.805	−2.070	−2.150	−2.300	−2.283	0.389
***t*****–bar test, N, T** = **(38, 29) Obs** = **1,064 Augmented by 1 lags (average)**							
Model 2. High income group countries	Lung cancer	−3.105	−2.040	−2.110	−2.230	−8.548	< 0.001
	Urban	−2.013	−2.040	−2.110	−2.230	−1.606	0.054
	Outdoor	−3.256	−2.040	−2.110	−2.230	−9.508	< 0.001
	Indoor	−3.256	−2.040	−2.210	−2.230	−9.508	< 0.001

In line with the test results obtained, appropriate panel unit root tests were applied. The null hypothesis H0, which states that there is not horizontal cross-section dependence in the series, is rejected. That is, words, the series have cross-sectional dependence. After this part of the research, secondary unit root tests that consider horizontal cross-section dependence will be applied.

Since the *t*-bar (CADF) statistic is more significant in absolute value than the given critical value at the 10% (cv10), 5% (Lcv5), and 1% (cv1) confidence levels, the series are stationary, except for Indoor Air Pollution variable (Table 3). Model 2 high-income group Urbanization variable is standing at 10% level. After determining the stationarity status of the variables, the model to be developed will be examined to determine whether it meets the basic assumptions of panel data. The first assumption to be examined within the basic assumptions is to determine whether there are any variables in the model that could lead to multicollinearity.

Multicollinearity Problem: These models must meet certain assumptions to obtain accurate results from the models developed within the scope of panel data. Models without meeting the fundamental assumptions are prone to errors and may yield inaccurate findings. Therefore, in the first stage, it should be tested whether there are variables that may cause multicollinearity problems. If a model has multicollinearity, the predictor coefficients may be miscalculated, as stated by Gujarati (33). Detecting this problem and not using variables that are highly correlated with each other in the same model will solve this problem. There are different tests and methods developed to reveal the existence of this problem. One of the most prominent of these methods is the calculation of Variance Inflation Factor (VIF) values of variables. The formula (1/1-*R*^2^) is used to calculate the VIF values of each variable (34). Although there is no consensus on acceptable VIF values and it is stated that these values can go up to 10, it is desired that these values be as low as 5 (35).

We obtained R square values by making each variable in the model a dependent variable and calculated VIF values with the help of the formula stated above. As can be seen from the information in Table 4, the most critical value of the variables is the coefficient of 5. The VIF values of the variables of the study are < 5 and these results show us that there is no multicollinearity. Therefore, there is no problem in continuing the analyzes with these variables. After this stage, it should be determined which estimation method is most suitable for the model. There are three basic modeling approaches in panel data modeling. These are traditional or classical model, random effects model and fixed effects model (36). Table 5 shows the results of the tests related to these models.

**Table 4:** VIF values of the variables.

	**Model 1 (middle income group lung cancer)**		**Model 2 (high income group lung cancer)**	
**Variables**	***R* ^2^**	**VIF value**	***R* ^2^**	**VIF value**
Lung cancer	0.26	1.35	0.17	1.20
Urban	0.59	2.43	0.41	1.69
Outdoor	0.34	1.51	0.29	1.40
Indoor	0.29	1.40	0.23	1.29

**Table 5:** Panel data model identification tests.

	**Model 1 (middle income group lung cancer)**		**Model 2 (high income group lung cancer)**	
	**Statistic value**	**Probability value**	**Statistic value**	**Probability value**
*F*-fixed effects	97.24	< 0.001	11.97	< 0.001
Hausman test	1.70	0.63	4.92	0.17

According to the findings in the Table 5, it is understood that the classical model is not appropriate for this model since the model has unit effects. After this finding, The Hausman test was applied to decide whether the approach valid for both models was the random effects or fixed effects approach. From the results of the Hausman test, fit was accepted that random effects approach is valid for both models (Table 5). Then, it should be checked whether there is autocorrelation in the model. When developing a panel data modeling, the models should have no autocorrelation. The models with means that the error terms of the variables are correlated with each other. If an autocorrelation problem is encountered in the model, the situation should be eliminated to obtain more accurate results. Our models were checked for autocorrelation using two different tests (Durbin-Watson and Baltagi-Wu LBI). Table 6 show the results of autocorrelation tests.

**Table 6:** Autocorrelation test results in models.

**Tests**	**Model 1 (middle income group lung cancer)**		**Model 2 (high income group lung cancer)**	
	**Statistic value**	**Probability value**	**Statistic value**	**Probability value**
Bhargava et al. Durbin-Watson	0.52	< 0.001	1.39	< 0.001
Baltagi-Wu LBI	0.73	< 0.001	1.43	< 0.001

From the results in Table 6, in both types of tests, the H0 hypothesis, which states the autocorrelation coefficients were equal to zero, was rejected. In line with the information in the literature, when these test values are < 2, it indicates an autocorrelation problem in the models. In these developed models, the test statistic values are considerably smaller than 2, indicating an autocorrelation problem in the model (Table 6). After testing other assumptions in the models, necessary robust correction tests will be applied by taking this problem into account. Another thing to consider is testing the existence of the heteroscedasticity problem. If the variance of the model changes due to changes in the units in the models, it is a sign that there is a changing variance problem. The modified Wald test is applied to check the presence this problem in both models. The results of the modified Wald test are shown in Table 7.

**Table 7:** Variance heteroskedasticity.

**Test**	**Model 1 (middle income group lung cancer)**		**Model 2 (high income group lung cancer)**	
	**Chi^2^**	**Probability value**	**Chi^2^**	**Probability value**
Modified Wald test	4,366.46	< 0.001	8,052.11	< 0.001

In the modified Wald test, the null hypothesis H0 is stated as no changing variance, and from the result in the Table 7, H0 is rejected. That is, both models have problem of heteroskedasticity which requires necessary corrections to be made (Table 7). Finally, the last assumption to be considered is determine whether the model as a whole has the cross-sectional dependence. We used three different tests to detect if the models have cross-section dependence. Results of these tests are shown in Table 8.

**Table 8:** Cross-sectional dependence test.

**Tests**	**Model 1 (middle income group lung cancer)**		**Model 2 (high income group lung cancer)**	
	**Statistic value**	**Probability value**	**Statistic value**	**Probability value**
Breusch-Pagan LM	3,687.35	< 0.001	1,089.57	< 0.001
Pesaran Scaled LM	138.28	< 0.001	277.01	< 0.001
Pesaran CD	13.27	< 0.001	42.85	< 0.001

According to the results of the three tests in Table 8, we can say that the models have cross-section dependence problem. Finally, as a result of all the tests we conducted to test the basic assumptions, we see that the problems of the autocorrelation, heteroscedasticity, and horizontal cross-sectional dependence in the model. Robust correction estimators will be used to overcome the negative effects of these issues in the models. The Driscoll and Kraay estimator, which eliminates the effects of the three problems mentioned in the robust correction estimators, will be used. Thanks to the Driscoll and Kraay robust correction test, the models will be free from the effects of these problems, and more robust estimator coefficients will be achieved.

The Table 9 shows the findings of the model developed for the middle-income countries' group. The dependent variable is lung cancer cases per 100,000 people. The independent variables are outdoor air pollution level, indoor air pollution level, and urbanization rate. The stationarity of all variables has been examined, non-stationary series have been differenced, and their stationary forms have been included in the model. From the *F*-statistic for Model 1 (middle-income countries), we can say that the model as a whole is significant. The *R* square value of 0.24 shows us that the model has a reasonable explanation percentage.

**Table 9:** Panel data results for models with Driscoll and Kraay standard error.

**Dependent variable: Model 1 (middle income group lung cancer), period: 1990–2019, horizontal section: 25, total number of observations: 725**				
**Variables**	**Coefficient**	**Drisc/Kraay standard error**	***t*** **-statistic value**	**Probability value**
Outdoor	0.282025	0.0635738	4.44	< 0.001
Indoor	4.157.320	1.783.419	0.23	0.818
Urban	0.1759048	0.0187327	3.52	0.002
*C*	−0.255227	0.0785676	2.09	0.003
*R*^2^: 0.24	*F*-statistic:11.97		Prob (*F*-Statistic): < 0.001	
**Dependent variable: Model 1 (high income group lung cancer), period: 1990–2019, horizontal section: 25, total number of observations: 725**				
**Variables**	**Coefficient**	**Drisc/Kraay standard error**	***t*** **-statistic value**	**Probability value**
Outdoor	0.5197649	0.0863754	6.02	< 0.001
Indoor	0.3734362	0.0547022	6.83	< 0.001
Urban	0.0615417	0.0026114	23.57	< 0.001
*C*	3.353.503	0.3826747	8.76	< 0.001
*R*^2^: 0.37	*F*-statistic: 22.56		Prob (*F*-Statistic): < 0.001	

When the effects of the independent variables on the dependent variable in the model are analyzed, firstly, it is seen that all variables have the increasing effect on the dependent variable. The coefficients of these variables show us that a one percent increase of the level of outdoor air pollution in a country will lead to a 0.28% increase in the rate of lung cancer. The variable for the indoor air pollution level of middle-income countries was insignificant in the model. Increasing in the rate of urbanization in the middle-income country group is also found to have an increasing effect on the dependent variable. In case of a 1% increase in the urbanization rate, it is predicted that there will be a 0.17% increase in lung cancer cases.

Table 9 also shows the findings of the model developed on lung cancer incidence for countries in the high-income country group. This model also suffers from autocorrelation, heteroscedasticity, and horizontal cross-sectional dependence problems, which are eliminated by using Driscoll and Kraay estimator. We see that this model is also significant at 1% significance level and has a higher *R* square value than other model (*R*^2^ = 0.37).

When the findings of the independent variables are analyzed, it is seen that all variables are positively related to the dependent variable. In other words, it has been determined that increases in the independent variables may also have increasing effect on the dependent variable. In the model, all variables are significant at the 1% level. It is predicted that a 1% increase in outdoor air pollution in developed countries (high-income group) would lead to a 0.51% increase in lung cancer cases. In the case of a 1% increase in indoor air pollution rate, it is estimated that there will be a 0.37% increase in the rate of lung cancer cases. In the case of a 1% increase in the rate of urbanization, it is predicted that there may be a 0.06% increase in lung cancer cases.

## 5 Discussion

In this study, the effects the variables of indoor air pollution, outdoor air pollution, and urbanization rate on the dependent variable of lung cancer and was examined. In this context, data from 25 middle-income and 38 high-income countries were obtained.

According to the results of the research, it was concluded that increases in the independent variables of indoor air pollution, outdoor air pollution, and urbanization rate have an increasing effect on the dependent variable of lung cancer. In the disease burden studies of Abbafati et al. (37) and Lim et al. (15), it was stated that indoor air pollution is among the critical disease burden risk factors. In addition, Field and Withers, Raaschou-Nielsen et al., and Wang et al. reported that indoor and outdoor air pollution were associated with lung cancer (14, 16, 20–38). In another study, Tseng et al. concluded that air pollution increases the risk of lung cancer in non-smokers (19). In a similar study, Raspanti et al. found that indoor air pollution increases lung cancer in individuals who have never smoked (23). Xie et al. reported that air pollution increases women's risk of lung cancer (39).

In this study, in middle-income countries, a 1% increase in outdoor air pollution increases the risk of lung cancer by 0.28%, and a 1% increase in urbanization rate increases the risk of lung cancer by 0.17%. On the other hand, the relationship between indoor air pollution and lung cancer was found to be insignificant in the model. According to the model, in upper-income group countries, a 1% increase in indoor air pollution increases the risk of lung cancer by 0.37%, a 1% increase in outdoor air pollution increases the risk of lung cancer by 0.51%, and a 1% increase in urbanization rate increases the risk of lung cancer by 0.06%. It is seen that the results obtained are in parallel with the literature.

A comprehensive prospective cohort study conducted by Liang et al. on 367,623 individuals based on UK Biobank participants concluded that exposure to air pollution causes an increased risk of lung cancer (40). Lee et al. conducted a study on patients diagnosed with lung cancer in Taiwan (41). They found that inhalation of fine particles containing heavy metals in polluted air or exposure to polluted air affects both lung cancer formation and mortality. Liu et al. investigated the long-term relationship between air pollution and lung cancer incidence in a study of 186,860 older adults aged 65 years and over diagnosed with lung cancer living in the United States between 2001 and 2016. In the study, long-term exposure to air pollutants (delicate particulate matter, nitrogen dioxide, and particle radioactivity) was significantly associated with lung cancer (42). Xue et al. revealed that outdoor and indoor air pollution affect lung cancer differently. It has been emphasized that workers exposed to particulate matter, asbestos, polycyclic aromatic hydrocarbons, and toxic metals in the environment resulting from industrial and vehicle exhausts as outdoor air pollution are more likely to develop lung cancer (43). It has been stated that indoor air pollution, cooking fumes, passive smoking, and radioactive products resulting from home decoration materials play a role in the development of lung cancer.

In a study conducted by Riaz et al. in England, it was reported that the incidence of lung cancer was higher in urban areas than in rural areas. In lung cancer survival results, it was concluded that there was a similarity between high-income levels in both urban and rural areas (44). In a study conducted by Guo et al. in China, it was reported that the incidence of lung cancer was higher in urban areas compared to rural areas. In addition, the incidence of lung cancer in middle and high-income levels was found to be lower than in low-income groups. In addition, it is reported that a low education level increases the incidence of lung cancer (45).

The sample of this research includes countries in the upper-income and middle-income groups (Table 10). The exclusion of the low-income group is one of the limitations of the research. The lack of data on the countries in the low-income group caused them to be excluded from the research. Another significant limitation of the study is the time dimension. The time dimension of the research is 1990–2019. Another limitation of the study is that the relevant data for periods other than the specified dates cannot be accessed. Data are obtained from the OECD database and are assumed to be accurate. The assumption that the data is accurate is also a significant limitation. The last limitation of the research is related to the variables included in the model. Lung cancer, the dependent variable, is also affected by other factors such as changes in smoking rates, life expectancy, and potential genetic influences (e.g., the difference between Caucasian and Asian populations). However, since the research aims to investigate the relationship between air pollution and lung cancer, other variables are not included in the modeling. In the model, the effect level of the variables that affect the dependent variable but are not included in the model is specified in the fixed variable.

**Table 10:** Countries included in the sampling scope of the research.

**Group of countries in the high income group**	**Group of countries in the middle income group**
Andora	Albania
Antigua and Barbuda	Argentina
Australia	Armenia
Austria	Azerbaijan
Bahamas	Brazil
Bahrain	Bulgaria
Barbados	Cuba
Belgium	Dominica
Canada	Dominican Republic
Chile	Ecuador
Denmark	Fiji
Estonia	Georgia
Finland	Iraq
France	Jordan
Germany	Lebanon
Greece	Libya
Greenland	Malaysia
Hungary	Mexico
Iceland	Moldova
Ireland	Panama
Israel	Peru
Italy	Romania
Latvia	Serbia
Luxembourg	South Africa
Malta	Turkey
Netherlands	
New Zealand	
Norway	
Poland	
Portugal	
Puerto Rico	
Qatar	
Slovenia	
Spain	
Sweden	
Switzerland	
United Kingdom	
United States	

## 6 Conclusions and recommendations

Today, air pollution is one of the leading health risks globally, and efforts are being made to protect against its effects. On the other hand, urbanization, which has been increasing continuously in recent years, poses significant health problems. With the increase in urbanization, the increase in the time spent indoors and the deterioration of air quality due to the dense population cause different health problems, especially lung health problems. Air pollution level is closely related to human health, and deterioration in air quality triggers many diseases. In this context, this study examines the relationship between air pollution levels and urbanization rates of middle-income and high-income countries and lung cancer, which is the most common cause of air pollution. In the model developed for the middle-income group, although the level of indoor air pollution has a high impact on lung cancer, it is insignificant due to the analysis. It was determined that increases in urbanization rate and outdoor air pollution levels might lead to a rise in lung cancer cases.

On the other hand, in the modeling developed for the developed country group, it is seen that the variable with the highest level of effect on lung cancer is the outdoor air pollution level. In parallel with the development of countries, it has been determined that the increase in industrial production wastes, in other words, worsening the air quality, may potentially cause an increase in lung cancer cases. Indoor air quality is also essential for human health; negative changes in this variable may negatively impact individuals' health, especially lung cancer.

In summary, in the context of both the studies in the literature and this research, long-term exposure to air pollution is associated with lung cancer and increases the risk of lung cancer. States have to take more stringent measures for the environment and air pollution to prevent this risk. At the same time, it is the responsibility of the state to ensure that individuals live in a clean and safe environment by enacting and implementing various protective laws and regulations that are more environmentally friendly. International organizations should promote preventive policies by creating more agenda and awareness. Air pollution is a global environmental problem that affects not only one country or region but the whole world. On the other hand, air pollution levels can trigger various diseases, especially lung-related diseases. The results can be compared by analyzing the relationship between the indicators related to air pollution levels of countries and different diseases. This research has a more comprehensive and macro-level perspective on lung cancer, one of the most important health problems of societies. On the other hand, it has been determined that indoor air pollutants and outdoor air pollutant particles have a direct effect on other health problems, especially lung cancer. In this way, in addition to raising social awareness, it also contributes to policy makers to develop preventive policies on the subject. For future studies, studies with panel data sets that include a wider time period or different country groups or variables such as smoking, genetic differences and life expectancy may be recommended.

## Data Availability

The original contributions presented in the study are included in the article/supplementary material, further inquiries can be directed to the corresponding author.

## References

[1] Cropper M Muller N Park Y Perez-Zetune V. The impact of the clean air act on particulate matter in the 1970s. J Environ Econ Manage. (2023) 121:102867. 10.1016/j.jeem.2023.102867

[2] Forouzanfar MH Alexander L Anderson HR Bachman VF Biryukov S Brauer M. Global, regional, and national comparative risk assessment of 79 behavioral, environmental and occupational, and metabolic risks or clusters of risks in 188 countries, 1990- 2013: a systematic analysis for the Global Burden of Disease Study 2013. Lancet. (2015) 386:2287–323. 10.1016/S0140-6736(15)00128-2 26364544 PMC4685753

[3] Brimblecombe P. Early Episodes. Air Pollution Episodes. London: World Scientific Publishing Europe Ltd. (2017). p. 11–26.

[4] Wang Y Zhong H. Mitigation strategies for controlling urban particulate pollution from traffic congestion: road expansion and road public transport. J Environ Manage. (2023) 345:118795. 10.1016/j.jenvman.2023.118795 37619390

[5] Khare M Nagendra SS. Artificial Neural Networks in Vehicular Pollution Modelling. Vol. 41. Berlin: Springer (2006).

[6] Po JY FitzGerald JM Carlsten C. Respiratory disease associated with solid biomass fuel exposure in rural women and children: systematic review and meta-analysis. Thorax. (2011) 66:232–9. 10.1136/thx.2010.147884 21248322

[7] Salvi SS Barnes PJ. Chronic obstructive pulmonary disease in non-smokers. Lancet. (2009) 374:733–43. 10.1016/S0140-6736(09)61303-9 19716966

[8] Ritchie H Spooner F Roser M. Causes of Death. (2018). Available at: https://ourworldindata.org/causes-of-death (accessed December 29, 2022).

[9] Pope CA 3rd Burnett RT Thun MJ Calle EE Krewski D Ito K. Lung cancer, cardiopulmonary mortality, and long term exposure to fine particulate air pollution. J Am Med Assoc. (2002) 287:1132–41. 10.1001/jama.287.9.1132 11879110 PMC4037163

[10] World Health Organization. Air Quality Guidelines. Global Update 2005. Particulate Matter, Ozone, Nitrogen Dioxide and Sulfur Dioxide. Copenhagen: World Health Organization (2005).

[11] Laden F Schwartz J Speizer FE Dockery DW. Reduction in fine particulate air pollution and mortality: extended follow up of the Harvard Six Cities study. Am J Respir Crit Care Med. (2006) 173:667–72. 10.1164/rccm.200503-443OC 16424447 PMC2662950

[12] Cardoso D Painho M Roquette R. A geographically weighted regression approach to investigate air pollution effect on lung cancer: a case study in Portugal. Geospat Health. (2019) 14:35–45. 10.4081/gh.2019.701 31099513

[13] Ritchie H Roser M. Outdoor Air Pollution. (2019). Available at: https://ourworldindata.org/outdoor-air-pollution (accessed December 29, 2022).

[14] Ritchie H Roser M. Indoor Air Pollution. Our World in Data (2013). Available at: https://ourworldindata.org/indoor-air-pollution (accessed December 29, 2022).

[15] Lim SS Vos T Flaxman AD Danaei G Shibuya K Adair-Rohani H. A comparative risk assessment of burden of disease and injury attributable to 67 risk factors and risk factor clusters in 21 regions, 1990-2010: a systematic analysis for the Global Burden of Disease Study 2010. Lancet. (2012) 380:2224–60. 10.1016/S0140-6736(12)61766-8 23245609 PMC4156511

[16] Raaschou-Nielsen O Andersen ZJ Beelen R Samoli E Stafoggia M Weinmayr G. Air pollution and lung cancer incidence in 17 European cohorts: prospective analyses from the European Study of Cohorts for Air Pollution Effects (ESCAPE). Lancet Oncol. (2013) 14:813–22. 10.1016/S1470-2045(13)70279-1 23849838

[17] Gariazzo C Binazzi A Alfò M Massari S Stafoggia M Marinaccio A. Predictors of lung cancer risk: an ecological study using mortality and environmental data by municipalities in Italy. Int J Environ Res Public Health. (2021) 18:1896. 10.3390/ijerph18041896 33669318 PMC7922734

[18] Wang L Sun W Zhou K Zhang M Bao P. Spatial analysis of built environment risk for respiratory health and its implication for urban planning: a case study of Shanghai. Int J Environ Res Publ Health. (2019) 16:1455. 10.3390/ijerph16081455 31022924 PMC6518356

[19] Tseng CH Tsuang BJ Chiang CJ Ku KC Tseng A Sen J. The relationship between air pollution and lung cancer in nonsmokers in Taiwan. J Thoracic Oncol. (2019) 14:784–92. 10.1016/j.jtho.2018.12.033 30664991

[20] Wang R Xu J Xu J Zhu W Qiu T Li J. MiR-326/Sp1/KLF3: a novel regulatory axis in lung cancer progression. Cell Prolif. (2019) 52:e12551. 10.1111/cpr.12551 30485570 PMC6495967

[21] Nemesure B Albano D Nemesure A. Short- and long-term survival outcomes among never smokers who developed lung cancer. Cancer Epidemiol. (2021) 75:102042. 10.1016/j.canep.2021.102042 34571392

[22] Field RW Withers BL. Occupational and environmental causes of lung cancer. Clin Chest Med. (2012) 33:681–703. 10.1016/j.ccm.2012.07.001 23153609 PMC3875302

[23] Raspanti GA Hashibe M Siwakoti B Wei M Thakur BK Pun CB. Household air pollution and lung cancer risk among never-smokers in Nepal. Environ Res. (2016) 147:141–5. 10.1016/j.envres.2016.02.008 26874046

[24] Soheylizad M Khazaei S Khazaei S Rezaeian S. Relation between lung cancer incidence and mortality rates with human development index and its components: a global ecological study. Iran J Cancer Prev. (2016) 9:5310. 10.17795/ijcp-5310

[25] Cohen AJ Pope CA. Lung cancer and air pollution. Environ Health Perspect. (1995) 103(Supp.8):219–24. 10.1289/ehp.95103s8219 8741787 PMC1518961

[26] Hu M Jiang C Meng R Luo Y Wang Y Huang MLF. Effect of air pollution on the prevalence of breast and cervical cancer in China: a panel data regression analysis. Environ Sci Pollut Res. (2023) 30:82031–44. 10.1007/s11356-023-28068-w 37318726

[27] Pang Z Xie B An Z And Wang L. Spatial and moderating effects of greenspace on the association between air pollution and lung cancer incidence. Appl Geograp. (2024) 164:1–11. 10.1016/j.apgeog.2024.103207

[28] Nesnow S Lewtas J. Mutagenic and carcinogeneic potency of extracts of diesel and related enviromental emissions: summary and discussion of results. Environ Int. (1981) 5:393–401. 10.1016/0160-4120(81)90093-333395953

[29] Huang YJ Lee PH Chen LC Lin BC Lin C Chan TC. Relationships among green space, ambient fine particulate matter, and cancer incidence in Taiwan: a 16-year retrospective cohort study. Environ Res. (2022) 212:113416. 10.1016/j.envres.2022.113416 35523280

[30] Kayyal-Tarabeia I Michael Y Lensky IM Blank M Agay-Shay K. Residential greenness and site-specific cancer: a registry based cohort of 144,427 participants with a 21-years of follow-up, Tel-Aviv district, Israel. Environ Res. (2022) 212:113460. 10.1016/j.envres.2022.113460 35561833

[31] Kasdagli MI Katsouyanni K de Hoogh K Lagiou P Samoli E. Associations of air pollution and greenness with mortality in Greece: an ecological study. Environ Res. (2021) 196:110348. 10.1016/j.envres.2020.110348 33127394

[32] Crouse DL Pinault L Balram A Brauer M Burnett RT Martin RV. Complex relationships between greenness, air pollution, and mortality in a population-based Canadian cohort. Environ Int. (2019) 128:292–300. 10.1016/j.envint.2019.04.047 31075749

[33] Gujarati DN. Basic Econometrics Mcgraw Hill. 4th ed. McGraw-Hill Companies (2003).

[34] O'Brien RM. A caution regarding rules of thumb for variance inflation factors. Qual Quant. (2007) 41:673–90. 10.1007/s11135-006-9018-6

[35] Acikgoz E Uygurturk H Korkmaz T. Analysis of factors affecting growth of pension mutual funds in Turkey. Int J Econ Fin Iss. (2015) 5:427–33.

[36] Beylik U Cirakli U Cetin M Ecevit E Senol O. The relationship between health expenditure indicators and economic growth in OECD countries: a Driscoll-Kraay approach. Front Public Health. (2022) 10:1050550. 10.3389/fpubh.2022.1050550 36478719 PMC9720277

[37] Abbafati C Abbas KM Abbasi-Kangevari M Abd-Allah F Abdelalim A Abdollahi M. Global burden of 87 risk factors in 204 countries and territories, 1990-2019: a systematic analysis for the Global Burden of Disease Study 2019. Lancet. (2020) 396:1223–49. 10.1016/S0140-6736(20)30752-2 33069327 PMC7566194

[38] Coyle YM Minahjuddin AT Hynan LS Minna JD. An ecological study of the association of metal air pollutants with lung cancer incidence in Texas. J Thoracic Oncol. (2006) 1:654–61. 10.1016/S1556-0864(15)30377-4 17409932

[39] Xie H Shao R Yang Y Cruz R Zhou X Tchounwou PB. Impacts of built environment on risk of women's lung cancer: a case study of China. Int J Environ Res Public Health. (2022) 19:7157. 10.3390/ijerph19127157 35742401 PMC9223189

[40] Liang H Zhou X Zhu Y Li D Jing D Su X. Association of outdoor air pollution, lifestyle, genetic factors with the risk of lung cancer: a prospective cohort study. Environ Res. (2023) 218:114996. 10.1016/j.envres.2022.114996 36481370

[41] Lee NW Wang HY Du CL Yuan TH Chen CY Yu CJ. Air-polluted environmental heavy metal exposure increase lung cancer incidence and mortality: a population-based longitudinal cohort study. Sci. Tot. Environ. (2022) 810:152186. 10.1016/j.scitotenv.2021.152186 34883183

[42] Liu CS Wei Y Yazdi MD Qiu X Castro E Zhu Q. Long-term association of air pollution and incidence of lung cancer among older Americans: a national study in the Medicare cohort. Environ Int. (2023) 181:108266. 10.1016/j.envint.2023.108266 37847981 PMC10691920

[43] Xue Y Wang L Zhang Y Zhao Y Liu Y. Air pollution: a culprit of lung cancer. J Hazard Mater. (2022) 434:128937. 10.1016/j.jhazmat.2022.128937 35452993

[44] Riaz SP Horton M Kang J Mak V Lüchtenborg M Møller H. Lung cancer incidence and survival in England: an analysis by socioeconomic deprivation and urbanization. J Thoracic Oncol. (2011) 6:2005–10. 10.1097/JTO.0b013e31822b02db 21892107

[45] Guo H Chang Z Wu J Li W. Air pollution and lung cancer incidence in China: who are faced with a greater effect? Environ Int. (2019) 132:105077. 10.1016/j.envint.2019.105077 31415963

